# A Comparative Analysis of the Standing Weight-Bearing Anteroposterior View Versus the Rosenberg View Radiographs and Their Correlation With Oxford Knee Scores in Grading Osteoarthritis of the Knee Joint

**DOI:** 10.7759/cureus.92105

**Published:** 2025-09-11

**Authors:** Maheshwar Lakkireddy, Mridupawan Barman, Deepak Kumar Maley, Srikanth Eppakayala, Deepankar Satapathy, Ranjith K Yalamanchili, Syed Ifthekar

**Affiliations:** 1 Orthopaedics, All India Institute of Medical Sciences, Bibinagar, Bibinagar, IND; 2 Orthopaedics and Trauma, All India Institute of Medical Sciences, Bibinagar, Bibinagar, IND; 3 Orthopaedic Surgery, All India Institute of Medical Sciences, Bibinagar, Bibinagar, IND

**Keywords:** anteroposterior view, joint space narrowing, knee osteoarthritis, oxford knee score, rosenberg view

## Abstract

Introduction: Knee osteoarthritis (KOA) is a chronic degenerative condition characterized by joint space narrowing (JSN), pain, and functional limitations, affecting a significant portion of the population in India. Radiographic evaluation (standing anteroposterior (AP) view - standard technique) is crucial for diagnosing and grading KOA. The aim of this study was to evaluate the efficacy of the Rosenberg posteroanterior (PA) view compared to the standard anteroposterior (AP) view in grading KOA and to estimate the correlation between joint space width (JSW), radiographic grading, and the Oxford knee score (OKS).

Materials and methods: A total of 384 knees (192 patients) aged 40 years and above, presenting with knee pain, were included in this cross-sectional study. Radiographs of the standing AP and Rosenberg views were taken, and the medial and lateral tibiofemoral JSW were measured digitally on calibrated images. KOA was graded using the International Knee Documentation Committee (IKDC) classification. Statistical analysis included Spearman’s rho correlation coefficient, with significance set at p<0.05.

Results: The mean medial JSW was 3.91±0.89 mm in the AP view and 3.52±1.02 mm in the Rosenberg view, with a strong correlation (p<0.0001). The mean lateral JSW was similar between views (5.88±0.47 mm in AP and 5.89±0.43 mm in Rosenberg), showing moderate correlation (p<0.0001). The Rosenberg view identified more severe KOA grades, with 41.9% of patients classified as IKDC Grade 3 or higher, compared to 27.6% in the AP view. Additionally, 18.2% of patients were reclassified to a more severe grade in the Rosenberg view, indicating its superior sensitivity in detecting early disease progression.

Conclusion: The Rosenberg view is more effective in detecting early and severe KOA, providing a more accurate assessment of disease severity for improved management.

## Introduction

Knee osteoarthritis (KOA) is a chronic degenerative joint condition that significantly impacts individuals' quality of life. It is characterized by the progressive joint space narrowing (JSN), loss of articular cartilage, osteophyte formation, subchondral sclerosis, and synovial inflammation. These pathological changes result in debilitating symptoms, including pain, stiffness, and functional limitations. KOA is a leading cause of disability worldwide, affecting both men and women, with women being disproportionately affected [1]. In India, the prevalence of KOA is approximately 28.7%, and it increases with age, making it a major public health concern, particularly in the aging population [1,2]. KOA can be classified into primary and secondary types, with primary osteoarthritis (OA) occurring without an identifiable cause, while secondary OA develops due to factors, such as post-traumatic events, rheumatoid arthritis, or joint infections [3,4].

Radiographic assessment is central to the diagnosis and grading of OA, offering a cost-effective and widely accessible means of evaluating the severity and progression of joint degeneration [5,6]. The standing weight-bearing anteroposterior (AP) radiograph has traditionally been the standard method for assessing KOA. However, recent studies have highlighted the benefits of the Rosenberg posteroanterior (PA) flexion view, which has been shown to be more sensitive in detecting early JSN, especially in the medial compartment of the knee [7,8]. The ability to detect early changes in joint space is crucial for early intervention and better management of the condition.

Grading KOA accurately using radiographs is essential for determining the appropriate treatment options [9]. These may range from conservative management strategies, such as physical therapy or pharmacological interventions, to more invasive surgical procedures such as high-tibial osteotomy (HTO) or total knee arthroplasty (TKA) in severe cases [10]. Early and precise grading of OA also helps in predicting the progression of the disease and determining the potential benefits of various therapeutic options [11]. Furthermore, correlating radiographic findings with clinical outcome measures, such as the Oxford knee score (OKS), provides a comprehensive understanding of the disease's severity and its impact on the patient’s functional abilities. The OKS is widely used to assess the clinical and functional status of individuals with KOA and to monitor the effectiveness of treatment interventions [12].

The aim of this study was to compare the efficacy of the Rosenberg PA view radiograph with the standing AP view radiograph in grading KOA. Specifically, the study focused on measuring the joint space width (JSW) in both radiographic views and grading tibiofemoral OA according to the International Knee Documentation Committee (IKDC) classification system. Additionally, the study examined the correlation between radiographic findings and the OKS, providing insights into the relationship between structural changes in the knee joint and the clinical and functional status of individuals with OA.

## Materials and methods

This cross-sectional study was conducted at the orthopedics outpatient department of All India Institute of Medical Sciences (AIIMS), Bibinagar, Telangana, India, between February 1, 2023, and August 1, 2024. The study was approved by the Institute Research Committee (IRC) and the Institute Ethics Committee (IEC) (AIIMS/BBN/IEC/DEC/2022/238-R). Informed consent was obtained from all participants in their preferred language before their inclusion in the study, ensuring compliance with ethical standards and guidelines for human research.

The study included patients aged 40 years or older who presented with knee pain. A consecutive non-randomized sampling method was used to recruit 384 knees (192 patients). The sample size was calculated based on an assumed 48% prevalence of the factor of interest, a 95% confidence interval, and 5% absolute precision, using the Statulator tool. Patients were excluded if they had inflammatory arthritic diseases, knee valgus deformity exceeding 10 degrees, or a history of significant knee trauma.

All participants underwent radiographic evaluation using both the Rosenberg (PA) view and standard standing AP view knee X-rays. The tibiofemoral JSW was measured in both the medial and lateral compartments digitally on the calibrated radiographic images. Radiographs were obtained using a standardized protocol, with the X-ray beam centered on the joint line and exposure parameters kept constant across all participants. Digital images were calibrated using an in-built scale provided by the radiology workstation to ensure accurate measurement of JSW. Measurements were performed by two independent observers, and the mean value was used for analysis to minimize interobserver variability. The International Knee Documentation Committee (IKDC) grading was applied to both views to assess knee joint integrity and the severity of knee arthritis. Clinical and functional status was evaluated using the OKS, a 12-item questionnaire that assesses pain, physical function, and other knee-related symptoms [13]. The OKS (Apppendix) was divided into four grading categories: Grade A (40-48 points), indicating satisfactory knee function; Grade B (30-39 points), indicating mild-to-moderate knee arthritis; Grade C (20-29 points), indicating moderate to severe knee arthritis; and Grade D (0-19 points), indicating severe knee arthritis.

Statistical analysis was performed using Statistical Product and Service Solutions (SPSS, version 26; IBM SPSS Statistics for Windows, Armonk, NY). Descriptive statistics, including frequencies and percentages, were used for categorical variables, while continuous variables were expressed as mean ± standard deviation. Spearman’s rho correlation coefficient was used to assess the relationship between the medial and lateral JSW measurements from the AP and Rosenberg radiographs. Statistical significance was set at p<0.05 for all analyses.

## Results

The demographic characteristics of the study population revealed a varied distribution across age groups, with the majority of participants falling within the 40-50 age group (162, 42.19%), followed by 51-60 years (136, 35.42%). A smaller percentage of participants were aged 61-70 years (66, 17.19%) and above 70 years (20, 5.21%). Regarding gender, a higher proportion of participants were females (240, 62.50%) compared to male participants (144, 37.50%). In terms of occupation, housewives comprised the largest group (136, 35.42%), followed by those in jobs (104, 27.08%), farmers (70, 18.23%), and business owners (68, 17.71%). A small number of participants were retired (4, 1.04%) or in service (2, 0.52%). The study also noted several comorbidities, with diabetes mellitus being the most prevalent (68, 17.71%), followed by hypertension (58, 15.10%) and hypothyroidism (40, 10.42%) (Table 1).

**Table 1:** Demographic characteristics Frequency and percentage were calculated for all variables.

Characteristic	Category	Frequency	Percentage (%)
Age Group	40-50 years	162	42.19
	51-60 years	136	35.42
	61-70 years	66	17.19
	>70 years	20	5.21
Gender	Male	144	37.50
	Female	240	62.50
Occupation	Housewife	136	35.42
	Job	104	27.08
	Farmer	70	18.23
	Business	68	17.71
	Retired	4	1.04
	Service	2	0.52
Comorbidities	Diabetes Mellitus	68	17.71
	Hypertension	58	15.10
	Hypothyroidism	40	10.42

Regarding knee pain and deformity characteristics, the study revealed that knee pain was equally distributed between the right and left knees, with 50% of participants reporting pain on each side. Knee deformity was present in 14.32% of the cases, while 85.68% did not exhibit deformities. A total of 119 (30.99%) participants had a 10-degree varus deformity, 12 (3.13%) participants had a 20-degree varus deformity, and 253 (65.89%) participants had no deformity. A range of motion (ROM) of 0-120 degrees was observed in 141 participants (36.72%), 0-130 degrees in 84 participants (21.88%), 0-100 degrees in 72 participants (18.75%), and 0-90 degrees in 40 participants (10.42%). The duration of knee pain varied, with 18.75% of participants experiencing pain for less than a year, 24.48% for one to two years, 34.38% for two to three years, and 25% experiencing pain for more than three years (Table 2).

**Table 2:** Knee pain and deformity characteristics Frequency and percentage were calculated for all variables.

Measure	Category	Frequency	Percentage (%)
Knee Pain Side	Right	192	50.00
	Left	192	50.00
Knee Deformity	Present	55	14.32
	Absent	326	85.68
Duration of Knee Pain	<1 Year	72	18.75
	1-2 Years	94	24.48
	2-3 Years	132	34.38
	>3 Years	96	25.00

**Figure 1:**
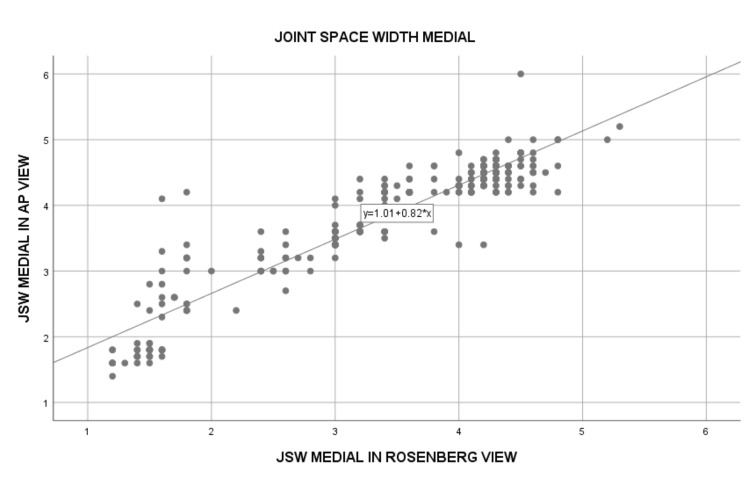
Simple scatter plot showing the medial joint space width (JSW) in AP view and Rosenberg view

**Figure 2:**
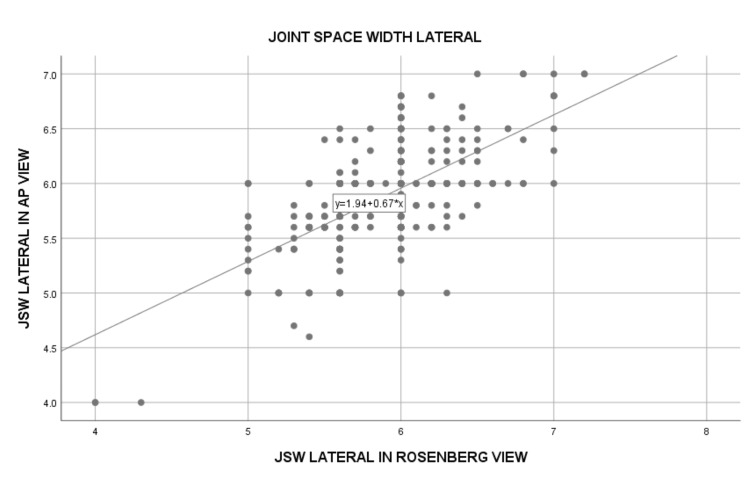
Simple scatter plot showing the lateral joint space width (JSW) in AP view and Rosenberg view

The comparison of JSW between the standing AP and Rosenberg views showed significant differences. The mean JSW in the medial compartment was 3.91 ± 0.89 mm in the AP view and 3.52 ± 1.02 mm in the Rosenberg view, with a strong positive correlation (Spearman's rho = 0.873; p<0.0001) (Figure 1). The lateral compartment JSW was slightly higher, with 5.88 ± 0.47 mm in the AP view and 5.89 ± 0.43 mm in the Rosenberg view. The correlation for the lateral compartment was moderate but still statistically significant (Spearman's rho = 0.527, p<0.0001) (Figure 2). These results suggest that both radiographic views provide valuable measurements, with the Rosenberg view showing greater sensitivity in detecting joint space narrowing, especially in the medial compartment (Table 3).

**Table 3:** Correlation between AP view and Rosenberg view for the medial and lateral joint space widths Spearman’s rho correlation coefficient was used to assess the relationship between the medial and lateral joint space width measurements from the AP and Rosenberg radiographs. Statistical significance was set at p<0.05.

Joint Space Width (mm)	AP View (Mean ± SD)	Rosenberg View (Mean ± SD)	Spearman's Rho	p-value
Medial	3.91 ± 0.89	3.52 ± 1.02	0.873	<0.0001
Lateral	5.88 ± 0.47	5.89 ± 0.43	0.527	<0.0001

The correlation between the OKS and the IKDC classification system was examined in this study. The findings revealed a strong association between the two measures, with higher OKS grades correlating with better IKDC grades (A and B). For instance, in the AP view, 89 participants were graded as A in both OKS and IKDC, while the Rosenberg view (RB) showed similar trends. A significant number of participants with lower IKDC grades (C and D) also had lower OKS scores, indicating inferior clinical outcomes. The correlation was statistically significant (p<0.0001), highlighting the potential of both tools in assessing the severity and impact of KOA on patients’ clinical and functional status (Table 4).

**Table 4:** Comparison between Oxford knee scores and IKDC scores AP = Anterioposterior view; RB = Rosenberg view; IKDC: International Knee Documentation Committee

Oxford Knee Score Grades	IKDC score							
	A		B		C		D	
	AP	RB	AP	RB	AP	RB	AP	RB
A	89	89	1	1	-	-	-	-
B	1	-	134	136	2	1	-	-
C	-	-	44	-	55	97	-	2
D	-	-	2	-	22	-	34	58
p-value	<0.0001							

## Discussion

This study underscores the significance of the Rosenberg view (PA flexion view) over the standard AP view in the early detection and grading of KOA. The demographic findings of our study are consistent with other studies, which highlight the age-related prevalence of KOA, with the majority of participants in the age group of 40-60 years. This correlates with the progressive nature of the disease, which worsens with age [2,14]. Additionally, the higher prevalence of KOA among females, as observed in our study, aligns with existing literature that suggests gender plays a significant role in the development of OA, possibly due to hormonal and biomechanical factors [15].

Our findings regarding JSW also provide compelling evidence for the superior sensitivity of the Rosenberg view in detecting joint space narrowing compared to the AP view. Previous studies have shown that the Rosenberg view is more effective in identifying early signs of OA, such as subchondral sclerosis and joint line narrowing, which are often missed in the AP view [16,17]. The strong correlation between JSW measurements in the Rosenberg view and the clinical evaluation via the OKS further supports its utility in the early diagnosis and management of KOA. Notably, the significant reduction in JSW in the Rosenberg view compared to the AP view highlights its potential as a more reliable diagnostic tool for detecting early-stage OA.

The ROM and deformity assessment also revealed interesting insights into the functional impairment caused by KOA. Our study found that a significant portion of patients exhibited reduced ROM, with the most common range between 0 and 120 degrees. This is consistent with the findings of Epskamp et al., who emphasized that KOA leads to stiffness and functional limitations that impact daily activities [18]. Moreover, the prevalence of deformities, such as varus alignment and flexion deformity, further indicates the advanced nature of the disease in many of the study participants. These findings underscore the importance of early intervention and accurate radiological assessment in preventing the progression of deformities and improving patient outcomes.

The discrepancy observed between the AP and Rosenberg views in the grading of KOA based on the OKS emphasizes the need for more refined imaging techniques in clinical practice. While both views showed good agreement in milder cases (Grade A), the Rosenberg view demonstrated better accuracy in identifying moderate to severe OA (Grades B and C), which is essential for determining appropriate treatment plans. The statistically significant differences between the two views further validate the Rosenberg view as a more sensitive imaging tool for the early detection of KOA. These findings are consistent with prior research that has highlighted the advantages of the Rosenberg view, particularly in identifying early radiological changes such as JSN, which may not be apparent in standard AP radiographs [2]. Therefore, incorporating the Rosenberg view into routine clinical practice could enhance the diagnostic accuracy and management of KOA.

This study was limited by a relatively small sample size and a single-center design, which may affect the generalizability of the findings. Additionally, inter-observer variability in radiographic interpretation was not assessed. The cross-sectional nature of the study also restricts the evaluation of disease progression over time.

## Conclusions

In cases where the OKS categorizes the patient's condition as mild (Grades A and B), either the AP view or the Rosenberg view may be employed for imaging. Conversely, if the OKS is classified as Grade C or D, it is advisable to obtain a Rosenberg view X-ray for better assessment. By following this protocol, healthcare professionals can ensure a more accurate assessment and, consequently, improve management strategies for OA.

## Appendices

**Table 5:** Oxford Knee Score (OKS)

1.	During the past 4 weeks…				
	How would you describe the pain you usually have from your knee?				
None		Very mild	Mild	Moderate	Severe
£		£	£	£	£

2.	During the past 4 weeks…				
	Have you had any trouble with washing and drying yourself (all over) because of your knee?				
No trouble at all		Very little trouble	Moderate trouble	Extreme difficulty	Impossible to do
£		£	£	£	£

3.	During the past 4 weeks…				
	Have you had any trouble getting in and out of a car or using public transport because of your knee? (whichever you tend to use)				
No trouble at all		Very little trouble	Moderate trouble	Extreme difficulty	Impossible to do
£		£	£	£	£

4.	During the past 4 weeks…				
	For how long have you been able to walk before pain from your knee becomes severe? (with or without a stick)				
No pain/More than 30 minutes		16 to 30 minutes	5 to 15 minutes	Around the house only	Not at all/pain severe when walking
£		£	£	£	£

5.	During the past 4 weeks…				
	After a meal (sat at a table), how painful has it been for you to stand up from a chair because of your knee?				
Not at all painful		Slightly painful	Moderately painful	Very painful	Unbearable
£		£	£	£	£

6.	During the past 4 weeks…				
	Have you been limping when walking, because of your knee?				
Rarely/ never		Sometimes, or just at first	Often, not just at first	Most of the time	All of the time
£		£	£	£	£

7.	During the past 4 weeks…				
	Could you kneel down and get up again afterwards?				
Yes, easily		With little difficulty	With moderate difficulty	With extreme difficulty	No, impossible
£		£	£	£	£

8.	During the past 4 weeks…				
	Have you been troubled by pain from your knee in bed at night?				
No nights		Only 1 or 2 nights	Some nights	Most nights	Every night
£		£	£	£	£

9.	During the past 4 weeks…				
	How much has pain from your knee interfered with your usual work (including housework)?				
Not at all		A little bit	Moderately	Greatly	Totally
£		£	£	£	£

10.	During the past 4 weeks…				
	Have you felt that your knee might suddenly 'give way' or let you down?				
Rarely/Never		Sometimes, or just at first	Often, not just at first	Most of the time	All of the time
£		£	£	£	£

11.	During the past 4 weeks…				
	Could you do the household shopping on your own?				
Yes, easily		With little difficulty	With moderate difficulty	With extreme difficulty	No, impossible
£		£	£	£	£

12.	During the past 4 weeks…				
	Could you walk down one flight of stairs?				
Yes, easily		With little difficulty	With moderate difficulty	With extreme difficulty	No, impossible
£		£	£	£	£
